# Susceptibility of Thymocytes for Infection by Chicken Anemia Virus is Related to Pre- and Posthatching Development

**DOI:** 10.1155/1992/52484

**Published:** 1992

**Authors:** Suzan H. M. Jeurissen, Marga E. Janse, Dirk J. van Roozelaar, Guus Koch, Gerben F. de Boer

**Affiliations:** Central Veterinary Institute Department of Virology P. O. Box 365 Lelystad 8200 AJ The Netherlands

**Keywords:** Thymocytes, chicken anemia virus, chicken, development

## Abstract

To investigate the age-dependent mechanism of susceptibility for chicken anemia virus (CAV) infection, we inoculated embryos and chickens of ages between day 9 of embryonic development and day 28 after hatching with CAV. Chicken embryos inoculated at days 9 and 11 of development showed no CAV-infected cells in the thymus, nor in other lymphoid organs. Many CAV-infected cells were detected in the thymic cortex of all chicken embryos inoculated at days 13 and 16 of development and of all chickens inoculated 1, 3, and 7 days after hatching. All embryos and chickens that contained CAV-infected cells in the thymus also contained CAV-infected cells in the bone marrow, but not in the bursa of Fabricius or the spleen. In chickens inoculated at days 14 and 21, only few CAV-infected cells were detected in the thymus, whereas these cells were not detected in thymi of 28-day-old inoculated chickens. Depletion of the thymic cortex was only detected in chickens inoculated from day 16 of embryonic development till day 21 after hatching. Only hematocrit values of the chickens inoculated 1 and 3 days after hatching were below normal. The rationale for the simultaneous susceptibility of cells of the T-cell lineage and cells of the erythrocyte lineage is discussed. As far as the thymus is concerned, the absence of clinical and microscopical signs of CAV infection in older chickens and the inability of CAV to infect embryos at days 9 and 11 of embryonic development may be caused by a lack of susceptible thymocytes. In view of the three waves of thymic precursor cells that populate the thymus during ontogeny, as described by Le Douarin and colleagues, we hypothesize that CAV only infects thymocytes derived from the second wave of precursor cells.


*Developmental Immunology*, 1992, Vol. 2, pp. 123–129
Reprints available directly from the publisher
Photocopying permitted by license only

© 1992 Harwood Academic Publishers GmbH
Printed in the United Kingdom

### Susceptibility of Thymocytes for Infection by Chicken Anemia Virus is Related to Pre- and Posthatching Development

SUZAN H. M. JEURISSEN,* MARGA E. JANSE, DIRK J. VAN ROOZELAAR, GUUS KOCH, and GERBEN F. DE BOER

*Central Veterinary Institute, Department of Virology, P. O. Box 365, 8200 AJ Lelystad, The Netherlands*

To investigate the age-dependent mechanism of susceptibility for chicken anemia virus (CAV) infection, we inoculated embryos and chickens of ages between day 9 of embryonic development and day 28 after hatching with CAV. Chicken embryos inoculated at days 9 and 11 of development showed no CAV-infected cells in the thymus, nor in other lymphoid organs. Many CAV-infected cells were detected in the thymic cortex of all chicken embryos inoculated at days 13 and 16 of development and of all chickens inoculated 1, 3, and 7 days after hatching. All embryos and chickens that contained CAV-infected cells in the thymus also contained CAV-infected cells in the bone marrow, but not in the bursa of Fabricius or the spleen. In chickens inoculated at days 14 and 21, only few CAV-infected cells were detected in the thymus, whereas these cells were not detected in thymi of 28-day-old inoculated chickens. Depletion of the thymic cortex was only detected in chickens inoculated from day 16 of embryonic development till day 21 after hatching. Only hematocrit values of the chickens inoculated 1 and 3 days after hatching were below normal. The rationale for the simultaneous susceptibility of cells of the T-cell lineage and cells of the erythrocyte lineage is discussed. As far as the thymus is concerned, the absence of clinical and microscopical signs of CAV infection in older chickens and the inability of CAV to infect embryos at days 9 and 11 of embryonic development may be caused by a lack of susceptible thymocytes. In view of the three waves of thymic precursor cells that populate the thymus during ontogeny, as described by Le Douarin and colleagues, we hypothesize that CAV only infects thymocytes derived from the second wave of precursor cells.

KEYWORDS: Thymocytes, chicken anemia virus, chicken, development.

### INTRODUCTION

Worldwide, chicken anemia virus (CAV) infection of newly hatched chickens causes considerable health problems in the poultry industry. CAV was first isolated in Japan by Yuasa et al. (1979). The virus contains a single-stranded circular DNA genome of 2.3 kb (Noteborn et al., 1991). CAV causes destruction of erythroblastoid cells in the bone marrow and thus severe anemia. In addition, CAV causes a transient depletion of the thymus cortex (Yuasa et al., 1979; Goryo et al., 1985). This depletion only concerns thymocytes; macrophages, stromal and epithelial cells can still be detected with immunohistochemical techniques (Jeurissen et al., 1989). The depletion of the cortex is supposed to cause an immunodeficiency leading to enhanced concurrent infections and to vaccination failures. In the field, only CAV infection of newly hatched chickens gives rise to these problems, whereas older chickens can be infected with CAV without any symptoms.

In this study, we investigate the susceptibility of chickens with ages between day 9 of embryonic development and day 28 after hatching for infection with CAV under specific-pathogen-free conditions. CAV-infected cells are visualized with monoclonal antibody CVI-CAV-85.1 (Noteborn et al., 1991), whereas depletion of lymphoid organs is visualized with monoclonal

*Corresponding author.

antibody HIS-C7, specific for leucocytes (Jeurissen et al., 1988), and monoclonal antibody CVI-ChT-74.1, specific for thymocytes and T lymphocytes (Noteborn et al., 1991).

### RESULTS

### CAV Infection of 1-day-old Chickens

In order to standardize CAV effects on lymphoid organs, we had to obtain precise knowledge into the common pathogenesis of a CAV infection. Therefore, 1-day-old chickens were inoculated with CAV and at days 3, 5, 7, 10, 14, and 21, thymus, bone marrow, spleen, and bursa of Fabricius were investigated. At day 3 after inoculation, CAV-infected cells were not detected. CAV-infected cells were first observed in the outer rim of the thymic cortex just beneath the capsule at day 5 after inoculation.

At day 7, many CAV-infected cells were detected in the entire thymic cortex (Fig. 1a), whereas the first signs of depletion of thymocytes from the outer rim of the cortex became visible. At this time point, the bone marrow contained equal numbers of HIS-C7-positive leucocytes and HIS-C7-negative erythrocytes. Staining with CVI-CAV-85.1 demonstrated large areas of CAV-infected cells.

At day 10, less CAV-infected cells were detected in the thymus than at day 7, whereas the cells were located near the corticomedullary junction. At this time point, the outer half of the cortex was depleted of thymocytes. In the bone marrow, some CAV-infected cells still were observed, whereas the number of HIS-C7-negative cells had decreased in comparison with the HIS-C7-positive cells.

At days 14 and 21, few or no CAV-infected cells were detected in thymus and bone marrow. At day 14, the whole thymic cortex was depleted of thymocytes (Fig. 1b), whereas in the bone marrow, HIS-C7-negative erythrocytes were absent. Only at this time point, hematocrit values had decreased from 34% to below 25%. At day 21, the thymic cortex was completely repopulated with thymocytes. The bone marrow contained a considerable, but still decreased number of erythrocytes at this time point. At none of the time points, were the effects of CAV observed on the thymic medulla. No chickens contained CAV-infected cells in bursa or spleen, whereas also no histological changes of these organs were observed. These results showed that 7 and 14 days after inoculation are characteristic time points during the pathogenesis of a CAV infection.

### CAV Infection of Chickens 3, 7, 14, 21, and 28 Days Old

We investigated the pathogenesis of CAV infections in chickens at different ages. In all chickens, the presence of CAV-infected cells was investigated in thymus, bone marrow, spleen, and bursa 7 days after inoculation, whereas depletion of cells was investigated at day 14 after inoculation.

In the groups of chickens inoculated at days 3 and 7 after hatching, many CAV-infected cells were detected in the entire thymic cortex and in the bone marrow. Fourteen days after inoculation, these groups demonstrated a depletion of the thymic cortex comparable to that of 1-day-old infected chickens. At this time point, the bone marrow contained few erythrocytes. The depletion of erythrocytes was also demonstrated by the hematocrit values that were below 25%.

In the groups of chickens inoculated at days 14 and 21, some CAV-infected cells were detected in the outer border of the cortex (Fig. 1c). In these groups, only the outer rim of the cortex was depleted of thymocytes, whereas the inner part of the cortex and the medulla seemed unaffected (Fig. 1d). In the bone marrow of chickens inoculated at day 14, small clusters of CAV-infected cells were detected after 7 days, but depletion of erythrocytes was not demonstrated.

After inoculation at day 28, CAV-infected cells were almost absent in thymus and bone marrow. In the chickens inoculated at day 28, no signs of a CAV infection could be detected in the thymus or the bone marrow. In all chickens examined, CAV-infected cells or histological changes were not detected in bursa and spleen.

### CAV Infection of Chicken Embryos at Days 9, 11, 13, and 16 of Development

Seven days after inoculation, the presence of CAV-infected cells was investigated in thymus, spleen, and bursa of Fabricius of all embryos, whereas only bone marrow of 16-day-old infected embryos was investigated. In both groups of six embryos inoculated at days 9 and 11 of development, only one embryo contained a few CAV-infected cells in the thymus. In both groups of six embryos infected at days 13 and 16 of development, all embryos contained some CAV-infected cells in the thymus (Fig. 1e). Four

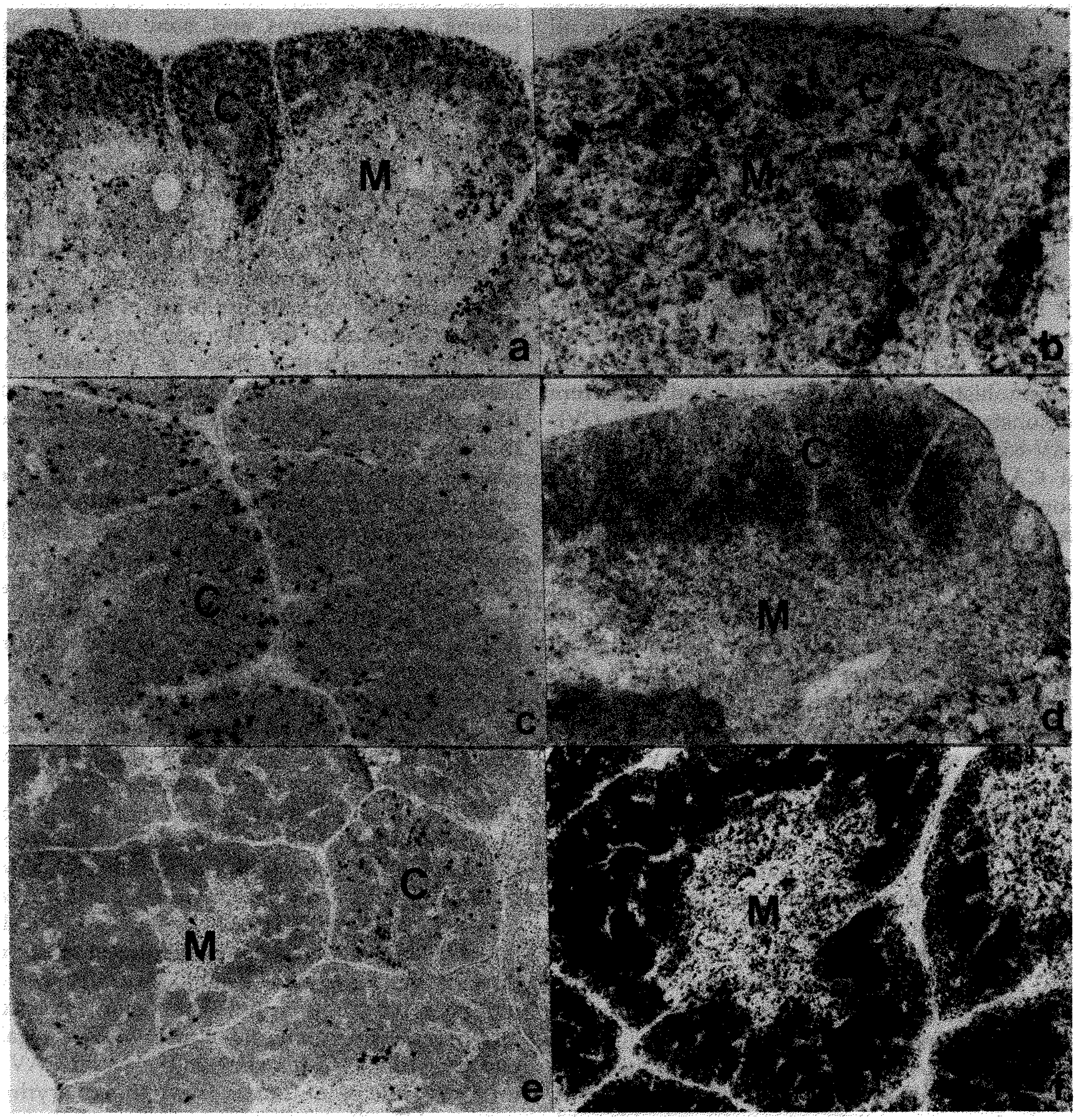

FIGURE 1. Thymus from embryonic and newly hatched chickens infected with CAV. C: cortex, M: medulla. (a) Seven days after CAV infection of 1-day-old chickens, many CVI-CAV-85.1-positive cells were detected in the cortex (×50). (b) Fourteen days after CAV infection of 1-day-old chickens, CVI-ChT-74.1 detected few thymocytes in the depleted cortex, whereas the medulla is unaffected (×100). (c) Seven days after CAV infection of 3-week-old chickens, some CVI-CAV-85.1-positive cells were detected in the outer border of the cortex (×50). (d) Fourteen days after CAV infection of 3-week-old chickens, only the outer rim of the cortex was depleted of CVI-ChT-74.1-positive thymocytes (×50). (e) Five days after CAV infection of embryos at day 16 of development, some CVI-CAV-85.1-positive cells were detected (×50). (f) Five days after CAV infection of embryos at day 16 of development, CVI-ChT-74.1-positive thymocytes of the cortex were less densely packed (×50).

of six embryos inoculated at day 16 contained CAV-infected cells in bone marrow. In spleen and bursa, no CAV-infected cells were detected. In the groups inoculated at days 9, 11, and 13 of development, immunohistochemical examination of the thymic cortex and medulla demonstrated no differences with age-matched controls. In all embryos inoculated at day 16 of development, the thymic cortex showed a less condensed structure of thymocytes (Fig. 1f), whereas the bone marrow seemed unaffected.

### DISCUSSION

To investigate the susceptibility of thymocytes for infection by CAV, we inoculated embryonic and newly hatched chickens with the virus and examined the effects 7 and 14 days later with immunohistochemistry. CAV-infected cells were demonstrated with monoclonal antibody CVI-CAV-85.1 (Noteborn et al., 1991). Previous results on CAV-infected cell lines indicated that this monoclonal is specific for a newly synthesized viral protein. Although the precise biological properties of CAV are unknown, the size and the nature of its genome and the epidemiological data give no indications for the occurrence of latent infections. We are therefore confident that all CAV-infected cells are recognized by this monoclonal antibody. The results concerning the presence and quantity of CAV-infected cells in the thymus are summarized in Table 1. CAV-infected cells were demonstrated in the thymic cortex of all chicken embryos inoculated at days 13 and 16 of development and of all chickens inoculated 1, 3, 7, 14, and 21 days after hatching. The number of CAV-infected thymocytes first increases with development and then gradually decreases. In addition, the bone marrow of embryos inoculated at day 16 of development and of chickens inoculated 1, 3, 7, and 14 days after hatching contained CAV-infected cells. Remarkably, spleen and bursa of CAV-inoculated chickens never contained CVI-CAV-85.1-positive cells, not even in the chicken embryos. These results coincide with those of Hoop and Reece (1991). Using a polyclonal anti-CAV antiserum, they detected specific staining in thymus and bone marrow, but not in spleen, bursa, and other organs. We therefore conclude that CAV cannot infect bursal lymphocytes, B lymphocytes, and peripheral T lymphocytes, but specifically infects erythropoietic cells and thymocytes. Other researchers, however, describe additional atrophy of the bursa during CAV infection (Goryo et al., 1989b; Lucio et al., 1990). As the depletion of lymphocytes from the bursa occurs 6 days after the appearance of lesions in the thymus and bone marrow, and because no intranuclear inclusions, which are specific for viral replication, were observed in bursal lymphocytes, Goryo and coworkers suggested that the lesions in the bursa may be induced by a different mechanism (Goryo et al., 1989b). In addition, the difference may be caused by the use of chickens that are not free of other viruses, such as infectious bursal disease virus or by the use of variant strains of CAV.

The simultaneous susceptibility of thymus and bone marrow cells for infection by CAV raises the question whether there is a relationship between the two cell populations. In wild birds, Kendall and Frazier (1979) have demonstrated that the thymus supports erythropoiesis to a great extent, whereas, also in the newly hatched chicken, some erythropoiesis is found in the thymus (M. Kendall, personal communication). The possibility exists that CAV only infects erythroblastoid cells in the thymus and that their destruction causes a cascade of events leading to further depletion of the cortex. Ultrastructural examination of thymus and bone marrow of chickens 6 and 10 days after inoculation demonstrated that both organs contained cells with electron-dense rings in the nucleus (Fig. 2). These electron-dense rings have been attributed to infection by CAV (Goryo et al., 1989a, 1989c; McNulty et al., 1990). In bone marrow, these infected cells obviously belong to the erythrocyte lineage (Fig. 2a); in the thymus, however, all infected cells are lymphocytes and virally infected erythroblasts cannot be detected at any time point (Fig. 2b). The CAV infection as observed by immuno-light and electron microscopy occurs simultaneously in both organs and therefore it seems very unlikely that CAV at the same time point infects precursors of erythrocytes and T cells in the bone marrow, as the consecutive migration to the thymus would cause a lag period.

Depletion of cortical thymocytes could be observed in all embryos and chickens inoculated between day 16 of development and day 21 after hatching. Remarkably, chickens inoculated at day 28 contained no CAV-infected cells in the thymic cortex. A possible reason might be that when chickens grow older, their ability to mount an immune response also increases. As this CAV infection reflects a primary viral infection, however, immunity and immunocompetence will not interfere with the susceptibility of the chickens. We therefore assume that the decreased infection of 28-day-old chickens is caused by a lack of susceptible thymocytes. The inability of CAV to infect chicken embryos at days 9 and 11 of development also seems to be due to a lack of susceptible thymocytes. In summary, only thymocytes that are located in the thymus between day 13 of embryonic development and day 21 after hatching are susceptible for infection with CAV.

Le Douarin and coworkers have elegantly demonstrated in quail-chicken chimeras that the thymus can accept three waves of stem cells during ontogeny (Le Douarin et al., 1980; Jotereau and Le Douarin, 1982). The first period of influx occurs around day 6.5, the second around day 12 of development, and the third around hatching. Within 4 to 7 days, each new wave of cells completely replaces the previous one. Based on our observations, we hypothesize that CAV infection is restricted to thymocytes derived from the second wave of precursor cells. These cells compose the thymus of a newly hatched chicken and, under normal conditions, they are gradually replaced by thymocytes of the third wave. At the moment that the thymus gets depleted by a CAV

TABLE 1
CVI-CAV-85.1-Positive Cells in the Thymus Detected 7 Days After Embryos and Chickens of Various Ages Were Inoculated with CAV

| | Inoculation at | | | | | | | | | |
|---|---|---|---|---|---|---|---|---|---|---|
| | ED9[a] | ED11 | ED13 | ED16 | d1 | d3 | d7 | d14 | d21 | d28 |
| Number[b] | 1/6 | 1/6 | 6/6 | 6/6 | 6/6 | 3/3 | 3/3 | 3/3 | 3/3 | 1/3 |
| Quantity[c] | +/− | +/− | +/− | + | ++ | ++ | ++ | + | + | +/− |

[a]ED: day of embryonic development.
[b]Number of chickens with CAV-infected thymocytes per total number of examined chickens.
[c]CAV-infected cells per positive thymus; +/−: few cells; +: some cells; ++: many cells.

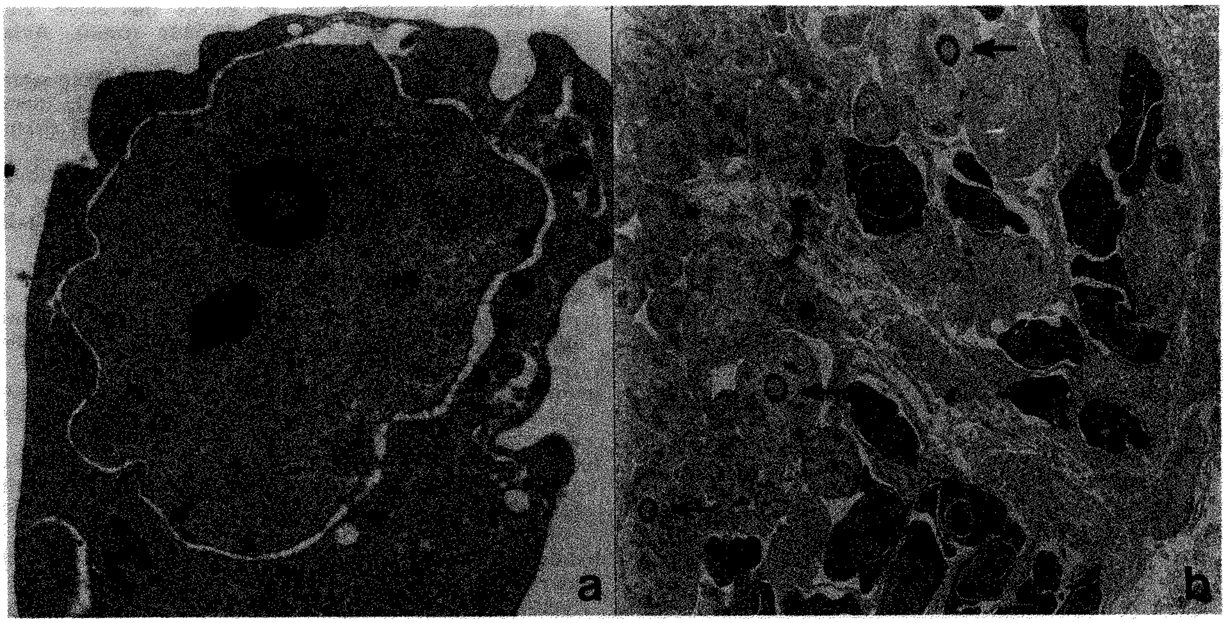

FIGURE 2. Ultrastructure of bone marrow and thymus from a 1-day-old chicken infected with CAV. (a) Six days after CAV infection, the bone marrow contained erythroblastoid cells with a characteristic electron-dense ring in the nucleus (×10,000). (b) Six days after CAV infection, the thymic cortex contained lymphocytes with a characteristic electron-dense ring in their nuclei (arrows) (×2500).

infection, the third wave precursor cells in the thymus will start to repopulate the thymus. Thus, the extremely fast repopulation of the cortex at day 21 may be explained. Interestingly, these third-wave precursor cells that repopulate the thymus and their newly formed progenitors are not susceptible to CAV. Further research is needed to elucidate the mechanism of this possible restriction of CAV.

### MATERIALS AND METHODS

### Animals

White Leghorn strain A (WLA) chickens were bred and kept under specific-pathogen-free conditions in Horsefall–Bauer isolators. Chicken embryos were inoculated into the blood vessels of the chorioallantois membrane at days 9, 11, 13, and 16 of embryonic development. At days 1, 3, 7, 14, 21, and 28 after hatching, chickens were inoculated into the M. pectoralis. The inoculation consisted of $10^5$ $TCID_{50}$ of a cell-free and chloroform-treated preparation of the Cux-1 strain of CAV that was propagated in MDCC-MSB1 cells (Goryo et al., 1987).

At various time points after inoculation, bone marrow, spleen, and bursa of Fabricius were frozen in liquid nitrogen, stored at −20 °C, and used for immunohistochemistry. Hematocrit values were determined of all chickens inoculated after hatching. At 6 and 10 days after inoculation of 1-day-old chickens, small tissue blocks of thymus and bone marrow were fixed and embedded for electron microscopy with conventional techniques. Ultrathin sections were examined in a Philips CM10 transmission electron microscope.

### Immunohistochemistry

Cryostat secretions of 8 $\mu$m thickness were picked up on slides, air dried, and fixed in pure acetone for 10 min. Sections were incubated with monoclonal antibodies in an appropriate dilution for 60 min. Monoclonal antibody CVI-CAV-85.1 is specific for CAV-infected cells (Noteborn et al., 1991), monoclonal antibody HIS-C7 is specific for leucocytes (Jeurissen ét al., 1988), and monoclonal antibody CVI-ChT-74.1 is specific for thymocytes and T lymphocytes (Noteborn et al., 1991). Slides were rinsed in phosphate-buffered saline (PBS, 0.01 M, pH 7.4) and covered with rabbit antimouse Ig peroxidase (Dakopatts, Glostrup, Denmark) in PBS containing 0.2% bovine serum albumin (BSA) for 60 min. To develop peroxidase activity, the slides were treated with 0.5 mg 3,3 -diaminobenzidine-tetrahydrochloride (DAB, Sigma Chemical Co., St. Louis, MO) per mL Tris-HCl buffer (0.05 M, pH 7.6) containing 0.01% $H_2O_2$. Control slides were incubated as described before, omitting only the first incubation step.

*(Received May 14, 1991)*

*(Accepted September 25, 1991)*

### REFERENCES

Goryo M., Hayashi S., Yoshizawa K., Umemura T., Itakura C., and Yamashiro S. (1989a). Ultrastructure of the thymus in chicks inoculated with chicken anaemia agent (MSB1-TK5803 strain). Avian Pathol. **18:** 605–617.

Goryo M., Sugimura H., Matsumoto S., Umemura T., and Itakura C. (1985). Isolation of an agent inducing chicken anaemia. Avian Pathol. **14:** 483–496.

Goryo M., Suwa T., Matsumoto S., Umemura T., and Itakura C. (1987). Serial propagation and purification of chicken anemia agent in MDCC-MSB1 cell line. Avian Pathol. **16:** 149–163.

Goryo M., Suwa T., Umemura T., Itakura C., and Yamashiro S. (1989b). Histopathology of chicks inoculated with chicken anaemia agent (MSB1-TK5803 strain). Avian Pathol. **18:** 73–89.

Goryo M., Suwa T., Umemura T., Itakura C., and Yamashiro S. (1989c). Ultrastructure of bone marrow in chicks inoculated with chicken anaemia agent (MSB1-TK5803 strain). Avian Pathol. **18:** 329–342.

Hoop R.K., and Reece R.L. (1991). The use of immunofluorescence and immunoperoxidase staining in studying the pathogenesis of chicken anaemia agent in experimentally infected chickens. Avian Pathol. **20:** 349–355.

Jeurissen S.H.M., Janse E.M., Ekino S., Nieuwenhuis P., Koch G., and De Boer G.F. (1988). Monoclonal antibodies as probes for defining cellular subsets in the bone marrow, thymus, bursa of Fabricius, and spleen of the chicken. Vet. Immunol. Immunopathol. **19:** 225–238.

Jeurissen S.H.M., Pol J.M.A., and De Boer G.F. (1989). Transient depletion of cortical thymocytes induced by chicken anaemia agent. Thymus **14:** 115–123.

Jotereau F.V., and Le Douarin N.M. (1982). Demonstration of a cyclic renewal of the lymphocyte precursor cells in the quail thymus during embryonic and perinatal life. J. Immunol. **129:** 1869–1877.

Kendall M.D., and Frazier J.A. (1979). Ultrastructural studies on erythropoiesis in the avian thymus. I. Description of cell types. Cell Tissue Res. **199:** 37–61.

Le Douarin N.M., Dieterlen-Lièvre F., and Oliver P.D. (1980). Ontogeny of primary lymphoid organs and lymphoid stem cells. Am. J. Anat. **170:** 261–299.

Lucio B., Schat K.A., and Shivaprasad H.L. (1990). Identification of the chicken anemia agent, reproduction of the disease, and serological survey in the United States. Avian Dis. **34:** 146–153.

McNulty M.S., Curran W.L., Todd D., and Mackie D.P. (1990). Chicken anemia agent: An electron microscopic study. Avian Dis. **34:** 736–743.

Noteborn M.H.M., De Boer G.F., Van Roozelaar D.J., Karreman C., Kranenburg O., Vos J.G., Jeurissen S.H.M., Hoeben R.C., Zantema A., Koch G., Van Ormondt H., and Van der Eb A.J. (1991). Characterization of cloned chicken anemia virus DNA that contains all elements for the infectious replication cycle. J. Virol. **65:** 3131–3139.

Yuasa N., Tanigushi T., and Yoshida I. (1979). Isolation and some properties of an agent inducing anaemia in chicks. Avian Dis. **23:** 366–385.

